# Assessment of Heavy Metal Contamination in Dust in Vilnius Schools: Source Identification, Pollution Levels, and Potential Health Risks for Children

**DOI:** 10.3390/toxics12030224

**Published:** 2024-03-19

**Authors:** Murat Huseyin Unsal, Gytautas Ignatavičius, Arunas Valiulis, Nina Prokopciuk, Roberta Valskienė, Vaidotas Valskys

**Affiliations:** 1 Institute of Biosciences, Life Sciences Center, Vilnius University, Saulėtekio Ave. 7, 10257 Vilnius, Lithuania; gytautas.ignatavicius@gf.vu.lt (G.I.); vaidotas.valskys@gmc.vu.lt (V.V.); 2 Clinic of Children’s Diseases, Institute of Clinical Medicine, Medical Faculty, Vilnius University, Antakalnio St. 57, 10207 Vilnius, Lithuania; 3 Department of Public Health, Institute of Health Sciences, Medical Faculty, Vilnius University, M. K. Čiurlionio St. 21, 03101 Vilnius, Lithuania; 4 Nature Research Centre, Laboratory of Ecotoxicology, Akademijos St. 2, 08412 Vilnius, Lithuania; roberta.valskiene@gamtc.lt; 5 Nature Research Centre, Laboratory of Climate and Water Research, Akademijos St. 2, 08412 Vilnius, Lithuania

**Keywords:** dust pollution, trace elements, indoor dust, dust exposure, environmental health risk, particulate matter, risk assessment, urban pollution, elemental analysis

## Abstract

The main objective of this study is to thoroughly evaluate the diversity and sources of heavy metals in the school environment. Specifically, this study examines the presence of heavy metals in the dust found and collected from 24 schools in Vilnius. Employing hierarchical cluster analysis, principal component analysis, and positive matrix factorization, we identified combustion-related activities as primary contributors to elevated metal concentrations, notably zinc, scandium, and copper, with PM2.5/PM10 ratios indicating a combustion source. They reveal significant differences in the levels of elements such as arsenic (4.55–69.96 mg/kg), copper (51.28–395.37 mg/kg), zinc, and lead, which are affected by both local environmental factors and human activities. Elevated pollution levels were found in certain school environments, indicating environmental degradation. Pollution assessment and specific element pairings’ strong positive correlations suggested shared origins or deposition processes. While this study primarily assesses non-carcinogenic risks to children based on a health risk assessment model, it acknowledges the well-documented carcinogenic potential of substances such as lead and arsenic. The research emphasizes the immediate necessity for efficient pollution management in educational environments, as indicated by the elevated hazard index for substances such as lead and arsenic, which present non-carcinogenic risks to children. This research offers important insights into the composition and origins of dust pollution in schools. It also promotes the need for broader geographic sampling and prolonged data collection to improve our understanding of pollution sources, alongside advocating for actionable strategies such as environmental management and policy reforms to effectively reduce exposure risks in educational settings. Furthermore, it aims to develop specific strategies to safeguard the health of students in Vilnius and similar urban areas.

## 1. Introduction

Dust, which is composed of solid particles in the form of fine powder (less than 100 µm), is commonly referred to as particulate matter (PM). It is highly polluting due to its ability to be easily transported through the air [1,2,3]. In urban areas, street dust plays a significant role in pollution. It is a complex mixture of particles that can contain various components such as organic matter, heavy metals, inorganic substances, mold spores, dander, and pollen. These particles can be resuspended into the air by vehicle movement and wind, thus becoming a major source of atmospheric pollution. They can settle on impermeable surfaces within cities, including roads and roofs [4,5,6,7]. A global study, such as the analysis of urban dust in six Mexican cities, emphasizes the widespread presence of heavy metal pollution and its associated health hazards, particularly for children [8].

According to Aguilera et al. [9], arsenic (As), cadmium (Cd), chromium (Cr), copper (Cu), mercury (Hg), manganese (Mn), nickel (Ni), lead (Pb), and zinc (Zn) are present in city street dust at concentrations higher than the median of the world soil background values. Pb, Zn, and Cu, while common in road transportation emissions, exhibit potential toxicity based on dose and exposure route, emphasizing the nuanced nature of environmental impact assessments. [10]. Consequently, high-traffic areas often exhibit elevated concentrations of heavy metals in street dust [1]. Poor vehicle maintenance, frequent stops, and slow driving below 30–40 km/h can result in excessive fuel consumption [11], negatively impacting both the environment and the physical health of drivers [12].

Indoor dust poses a significant concern for individuals who work, live, or spend a majority of their time indoors. This dust is a combination of particulate matter derived from both interior and exterior sources, and it can accumulate within indoor environments. Indoor dust serves as a notable source of metal exposure for people and this dust originates from various internal sources, including cooking, smoking, sweeping, wall erosion, rubber carpet materials, painting, building and furniture materials, consumer products, and other interior activities. On the other hand, external pollution sources contribute to indoor dust through the infiltration of emissions from traffic, auto repair, welding, waste burning, playground dust, and so on [2,3]. Comparative studies of indoor dust pollution across various global locations reveal significant variations in heavy metal concentrations. For instance, studies show elevated levels of Cr, Cu, Zn, Pb, and Fe in indoor dust from Malaysia, Iraq, Hong Kong, and Nigeria, among others [2,3,13,14,15]. Such variations are indicative of the widespread and diverse nature of indoor dust pollution, necessitating a deeper understanding of its sources and impacts on human health. Indoor dust in schools can have a significant impact on the health of children who study in classrooms. Children may be exposed to these heavy metals present in indoor dust through various routes such as inhalation, direct consumption of contaminated soils or food, and skin contact with polluted school materials [16]. Children are more vulnerable to the effects of heavy metals than adults. This vulnerability arises due to their behaviors, like hand-to-mouth contact, crawling activities, and their faster respiratory rate. These factors increase the likelihood of children ingesting heavy metals present in dust and inhaling more contaminated air compared to adults; also, As, Cd, Cr, and Pb are common environmental contaminants that might cause cancer as well as development disorders [17]. Although there has been much research on the worldwide presence of heavy metal pollution in urban environments, specifically in street dust as well as indoor dust, there is a significant lack of awareness regarding this matter in Lithuanian research, particularly in the context of schools.

The primary contribution of this work is in its thorough examination of metal contamination in indoor dust within schools in Vilnius, a topic that has been rarely explored in Lithuanian research. The lack of research in local studies provides a distinct chance for our investigation to clarify the origins and composition of indoor dust pollution in educational environments, which is an important issue that is frequently disregarded. Our hypothesis suggests that the indoor dust found in these schools has increased concentrations of heavy metals, which originate from a diverse range of both external and internal sources of pollution. 

The objective of our study is to not only measure these levels but also investigate their possible non-carcinogenic impacts on children. Our research aims to bring attention to the overlooked problem of long-term dust pollution in schools in Lithuania, specifically in the Vilnius region. We seek to provide new insights into the impact of this pollution on the health and safety of students in this area.

## 2. Materials and Methods

### 2.1. Sample Collection and Analysis

In various articles, different methodologies have been employed to collect dust samples from various locations. Some studies utilized vacuum cleaners and their bags as the collection method [13,18,19,20]. Other authors collected dust samples from multiple areas such as classroom floors, windowsills, playgrounds, balconies, doorsteps, stairs, entryways, fans, air conditioner filters, bookshelves, wall corners, desks, chairs [2,3,21,22,23].

**Figure 1:**
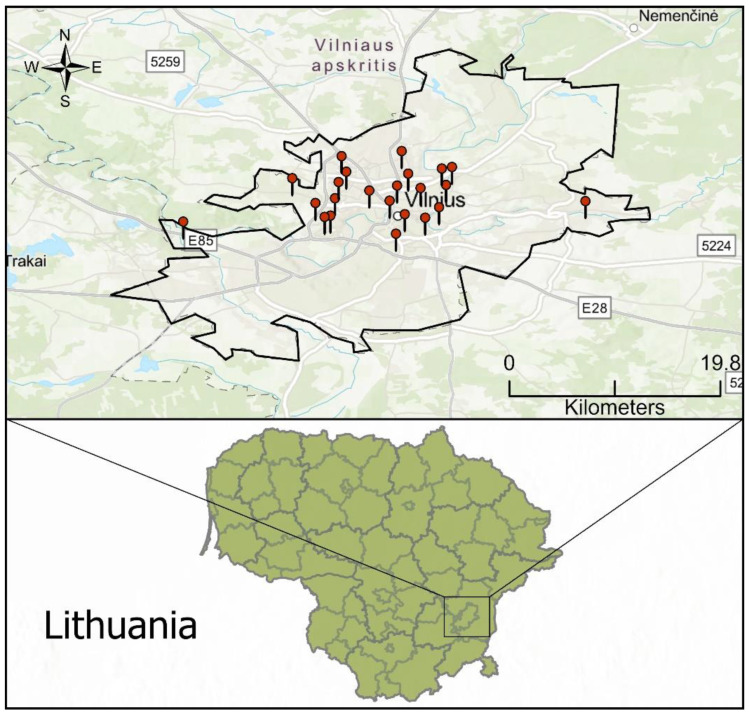
Geographical location and the area where the study was conducted.

In 2022, our research team collected dust samples from 24 schools (Figure 1) that were chosen based on specific criteria. Although most of the schools included in our study are located in the central area of Vilnius, a few of them are placed outside the city center, ensuring a full representation of different geographic positions in our research.

The process of selection offered priority to schools located near well-identified pollution sources, considering that Vilnius does not have major industrial contributors to pollution. The main focus of our criteria was on the heating plant, highways and railway infrastructure, which are the largest contributors to pollution in the city, and all the schools included in this study are located within a maximum proximity of 250 m from roads, highways, and railways according to their locations. Vilnius mostly relies on vehicular traffic for transportation, resulting in a wide range of transportation frequencies. In 2021, the automobile ownership rate in Vilnius was roughly 450.89 cars per 1000 inhabitants. This calculation is based on the presence of 365,577 cars and a population of 810,797 in the city [24]. Although there are no data on the exact transportation frequencies for each school, the high proportion of car ownership implies that there is a considerable amount of daily vehicular activity in the city. This information is important for assessing the potential exposure to environmental elements around schools.

We specifically chose schools built between 1930 and 2012 to ensure a diverse range of building ages and histories. This historical period includes various architectural eras, which mirrored the evolution of construction materials and methodologies throughout the years. The majority of these institutions have undergone renovations at different periods, which may impact the composition of interior dust. The selection criteria were designed to represent a comprehensive representation of dust accumulation in Vilnius, taking into account various architectural eras and maintenance approaches. The samples were obtained from areas typically overlooked by cleaners, like the space behind radiators, the top of bookcases, corners, windowsills, and inaccessible parts of gymnasiums. Our main focus was on the accumulation of dust over a long period of time. 

The collected dust samples were analyzed using Niton XL2 XRF Analyzer (XRF) spectrometry by Thermo Fisher Scientific (Waltham, MA, USA) [9,25]. Prior to analysis, the samples were prepared by breaking them into smaller pieces and mounting them on a sample holder. To ensure the integrity of our analysis, it was crucial that the capsules be clean and free from any contaminants that could interfere with the results. Each time the device was activated, calibration and system checks were conducted using standard samples with established concentrations. The apparatus was exclusively operated within a laboratory stand, setting the analysis duration to 600 s to maximize accuracy through exposure to three distinct characteristic energy lines. The accuracy of chemical element analysis varied, ranging from 10% for elements such as Cr, Cu, Zn, Zr, Sr, Rb, Mn, Fe, Ti, to 20% for As, Pb, Cd. Additionally, the device underwent inter-calibration with the SPECTRO XEPOS (SPECTRO Analytical Instruments GmbH, Kleve, Germany) energy dispersive X-ray fluorescence (ED-XRF) spectrometer at the Lithuanian Geological Survey, ensuring high measurement accuracy. XRF’s non-destructive nature (no acid treatment) allowed for the reuse of samples in multiple devices. However, results indicated up to a 20% significant systematic discrepancy. XRF is a commonly used method for analyzing the elemental composition of samples [26]. XRF spectrometry offers advantages such as element-specific detection and eliminates the need for the pre-treatment of the samples [25]; this technology, when used with adequate sample preparation and understanding of its limitations, can provide precise and reliable results as a valuable complement to laboratory analyses in the field of geochemical and environmental analysis, such as sample preparation differences, moisture content, matrix effects, and analytical interferences [27,28,29,30]. Mercury concentrations analyzed with the Niton XL2 Analyzer and SPECTRO XEPOS were undetectable; hence, analyses were limited to elements consistently detected by both instruments.

### 2.2. Pollution Assessment

#### 2.2.1. Geo-Accumulation Index (I_geo_)

The Geo-Accumulation Index (I_geo_) was initially introduced by Müller for evaluating metal concentrations in the 2-micron fraction of sediments. This index utilizes international standard shale values as a reference [31].

(1) $$I_{geo}={log}_{2}\frac{Cn}{Bn\times 1.5}$$

The concentration of a specific element in dust is denoted as Cn. The constant value of 1.5 is used to account for natural variations in element content and to detect even minimal anthropogenic influences. The geochemical background value is represented as Bn. Müller classified the Geo-Accumulation Index into seven classes, ranging from class 0 to class 6. The highest class, class 6, signifies an enrichment factor at least 100 times higher than the background values [31].

#### 2.2.2. Contamination Factor

The contamination factor (CF) is a method used to assess the level of contamination of indoor dust by a particular metal. It is calculated using the following equation [32]:

(2) $$CF=\frac{C_{Sample}}{C_{Background}}$$

The CF provides a quantitative measure of the extent to which the concentration of a specific metal in indoor dust deviates from the background concentration. The background value of trace elements in the Earth’s crust is denoted as C_Background_, while the concentration of the elements found in the samples is represented by C_Sample_. Background values for indoor and outdoor dust have not been established in this investigation. Alternatively, Vilnius and global background values for soils have been used [8]. The contamination factor (CF) can be classified as follows: CF < 1 indicates low contamination, CF between 1 and 3 represents moderate contamination, CF between 3 and 6 indicates considerable contamination, and CF greater than 6 suggests very high contamination [32]. According to studies [8,32], these categories are founded on accepted environmental research standards and offer a methodical way to assess the degree of contamination in urban dust.

### 2.3. Modified Degree of Contamination 

Modified degree of contamination (mCd) is a global contamination index that evaluates the degree of contamination of sediments, integrating all the toxic metals evaluated in the ecosystem [33]. mCd quantifies the absolute degree of contamination in a soil sample by dividing the sum of the contamination factors (Cf) of selected metals by the total number of measured metals (n). We used this approach and implemented it for our dust samples. This approach provides an average total value for various contaminants. The mCd is further classified into seven different classes to categorize the level of contamination [34].

The modified degree of contamination (mCd) is categorized into different levels of contamination: uncontaminated to very low (mCd ≤ 1.5), low (1.5 < mCd ≤ 2), moderate (2 < mCd ≤ 4), high (4 < mCd ≤ 8), very high (8 < mCd ≤ 16), extremely high (16 < mCd ≤ 32), and ultra-high (mCd > 32) [33].

(3) $$mCd=\frac{\sum_{i=1}^{n}Cf}{n}$$

### 2.4. Pollution Load Index

The Pollution Load Index (PLI) is a measure that assesses the overall pollution load resulting from the presence of hazardous metals at a specific site. The PLI is calculated by considering the contamination factor (CF) for each element present. CF represents the degree of contamination for each individual element.

By calculating the PLI for a particular location, information about the cumulative pollution load caused by all the hazardous metals can be obtained. When the PLI is less than 1, it indicates that there is no pollution present at the site. A PLI value of 1 suggests that only baseline levels of pollutants are present, implying minimal pollution. However, if the PLI exceeds 1, it indicates that the quality of the site has deteriorated due to pollution.

This methodology for assessing pollution load using the PLI, based on CF values, was discussed in a study conducted by Gope et al. [32] in 2017.

(4) $$PLI\;for\;a\;site={\left({CF}_{1}\times {CF}_{2}\times \ldots \times {CF}_{n}\right)}^{\frac{1}{n}}$$

(5) $$PLI\;for\;a\;zone={\left({PLI}_{site\;1}\times {PLI}_{site\;2}\times \ldots \times {PLI}_{site\;n}\right)}^{\frac{1}{n}}$$

### 2.5. Enrichment Factor

The enrichment factor (EF) is used to assess anthropogenic contaminant deposition on surface soil. It compares the concentration of a metal of interest to that of a stable reference element [35]. For EF calculations, we used Fe. Fe has been used by many authors working on marine and estuarine sediments [31,35]. EF values between 1 and 3 suggest natural weathering processes, while values above 3 indicate significant contributions from non-crustal sources like pollution. EF provides a measure of dust contamination, with values below 1 indicating no enrichment and values above 50 indicating extremely severe enrichment [31].

(6) $$EF=\frac{{[Cx/Cref]}_{Sample}}{{[Cx/Cref]}_{Background}}$$

### 2.6. Health Risk Assessment Model

The models used in this study to assess the risk of toxic metal exposure in school dust in adults and children are based on models developed by the US Environmental Protection Agency. The target receptors, primarily adults who work at school and children, are exposed via the following primary pathways: direct ingestion of dust (D_ing_), inhalation of dust particles through mouth and nose (D_inh_), dermal contact absorption (D_der_) (Table 1) [36].

(7) $$D_{ing}=C\times \frac{IngR\times EF\times ED}{BW\times AT}\times CF$$

(8) $$D_{inh}=C\times \frac{IngR\times EF\times ED}{PEF\times BW\times AT}$$

(9) $$D_{dermal}=C\times \frac{SL\times SA\times ABS\times EF\times ED}{BW\times AT}\times CF$$

The hazard quotient (HQ) and hazard index were used to evaluate the non-carcinogenic effects of metals (HI). A heavy metal’s HQ is calculated by dividing its ADD by its reference dose (RfD) for the same exposure pathway (s). The reference dose (RfD) (mg/kg day) (Table 2) is the highest daily dose of a metal from a particular exposure pathway, for both adults and children, that is thought not to significantly increase the risk of adverse effects on sensitive people over the course of their lifetime. It is assumed that there will not be any negative health impacts if the ADD is less than the RfD, HQ ≤ 1, but if the ADD surpasses the RfD, HQ ≥ 1, it is expected that there will be negative health effects. The hazard index (HI) is the total risk of a single non-carcinogenic factor through all three paths of exposure. The value of HI ≤ 1 indicates that there is no risk of non-carcinogenic effects, whereas HI ≥ 1 suggests that there is a chance of negative health impacts, and that chance grows as HI values rise [39].

(10) $$HI=\sum {HQ}_{i}=\sum \frac{{ADD}_{i}}{{RfD}_{i}}$$

### 2.7. Geospatial Mapping, Statistical Analysis and Data Computation

The Python programming language was used to perform statistical analyses and data computations. The geographic mapping software, ArcGIS Pro 10.8.1, was used to study and show the distribution of particulate matter (PM) throughout Vilnius. Maps were created using the Inverse Distance Weighted (IDW) interpolation approach, which relied on the PM concentration values. We chose IDW due to its capacity to precisely depict spatial fluctuations in PM concentrations, since it utilizes a linear combination of data points weighted inversely according to their distance. This method is especially efficient in emphasizing regions with significant pollution in close proximity to the selected schools, thus enabling a more accurate assessment of the risk linked to PM exposure. 

Principal component analyses (PCA) are a common method to reduce data and to identify a few latent factors (principal components, PCs) that capture the relationships among observed variables. A PCA can transform a set of correlated variables into a smaller set of orthogonal factors, facilitating the interpretation of a complex multidimensional system by showing the correlations among the original variables [40]. Hierarchical Clustering Analysis (HCA) is a technique that applies to data analysis tasks. The cluster analysis required standardizing the values with z-scores first, and then calculating the Euclidean distances among the heavy metal values. The hierarchical clustering used Ward’s method as the linkage criterion [41]. It aims to identify clusters of data points that share common characteristics or features. HCA can use different measures of similarity or dissimilarity to determine how close or far apart the data points are. The algorithm starts with treating every data point as an individual cluster. Next, in every iteration, it combines the two clusters that have the smallest distance or similarity measure between them. HCA can be visualized using dendrograms. A correlation matrix is a concise and informative way to summarize the linear associations among multiple variables in a dataset. It is a square matrix with each element showing the correlation coefficient between a pair of variables, ranging from −1 to 1. A positive coefficient indicates a positive linear relationship, while a negative coefficient indicates a negative linear relationship [3]. Clusters were determined by a k-means clustering algorithm and we used elbow and silhouette methods to understand optimal clusters. The silhouette coefficient varies from −1 to 1, and a model with a higher silhouette coefficient typically has more cohesive clusters [42].

A multivariate factor analysis tool called positive matrix factorization (PMF) was utilized to identify the sources of heavy metal pollution. This efficient analysis method involved breaking down the matrices of sample concentration figures into factor profile matrices and factor influence matrices. The sources of pollution were determined by analyzing the resulting profiles [43]. The PMF model was analyzed using EPA PMF 5.0 and was applied in this study to identify the origins and spatial distribution of metals in the soil. Initially, the model was set up with 3, 4, and 5 factors, and a random seed number was selected from 20 iterations to begin the analysis. The determination of the optimal number of factors was based on finding the lowest and most reliable Q true value. The search for an appropriate residual matrix E involved finding the minimum Q value, which helped establish the suitable number of factors. The PMF analysis resulted in the optimal output, providing the lowest Q value [34,44,45].

## 3. Results and Discussion

### 3.1. Heavy Metal Concentrations in School Environments

**Figure 2:**
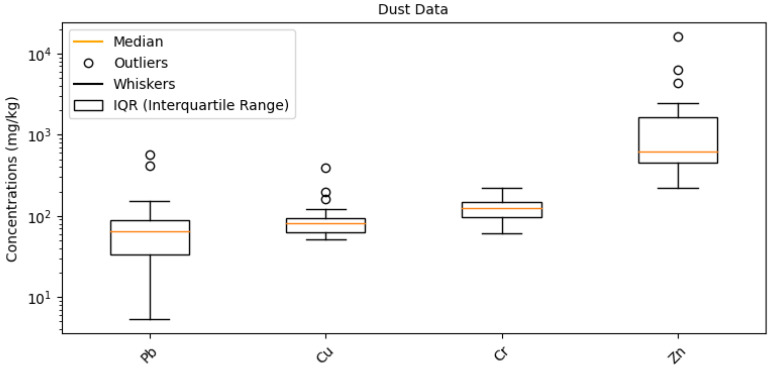
Distribution of Cu, Zn, Pb and Cr.

Diverse metal concentrations were found in the dust samples we analyzed from 24 schools in Vilnius, a city with a high traffic density but no direct industrial sources. Important discoveries include a notable variation in elemental concentrations, indicating different sources of contamination, such as As, Cu, Zn, and Pb. In contrast to As, Cu concentrations showed greater fluctuation, ranging from 51.28 mg/kg to 395.37 mg/kg. This variation, as seen in our comprehensive information (see Table S1 and Figure 2), is consistent with worldwide trends found in comparable settings. The variations in element concentrations between schools highlight the diversity of pollution sources in urban environments and the impact of local environmental variables.

Comparative analysis using Table S2 in the Supplementary Materials, which presents metal concentrations from various global indoor and outdoor environments, reveals interesting contrasts. For example, the As concentration in our samples ranged from 4.55 mg/kg to 69.96 mg/kg, while in South Africa’s School B it was 0.78 mg/kg and in Sydney 17.6 mg/kg. Such comparisons underline the influence of geographical and environmental factors on metal levels in dust, providing a broader context for understanding the results from Vilnius schools.

### 3.2. Contamination Factor, Modified Contamination Factor and Pollution Load Index Values

**Figure 3:**
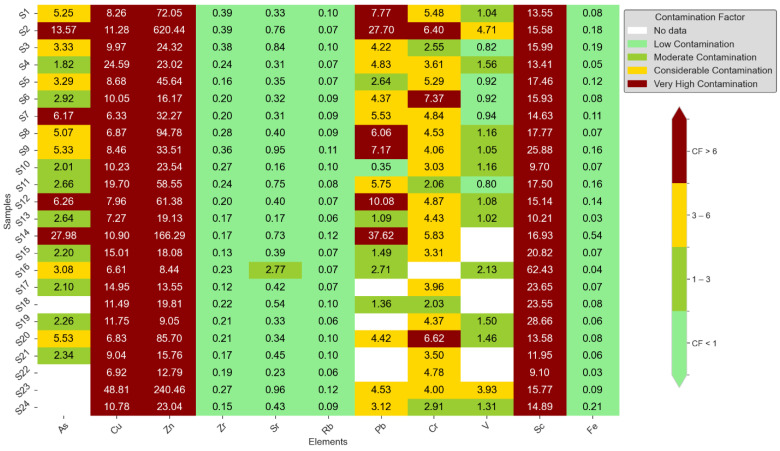
Results of contamination factor.

**Figure 4:**
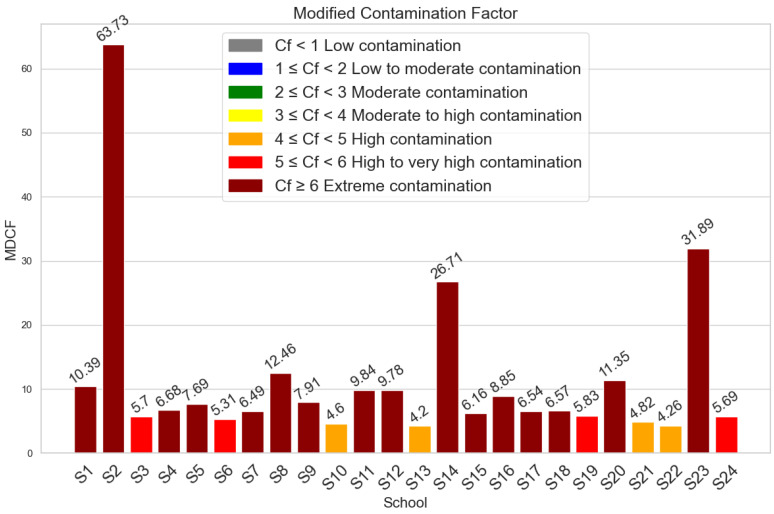
Modified contamination factor results of samples schools.

**Figure 5:**
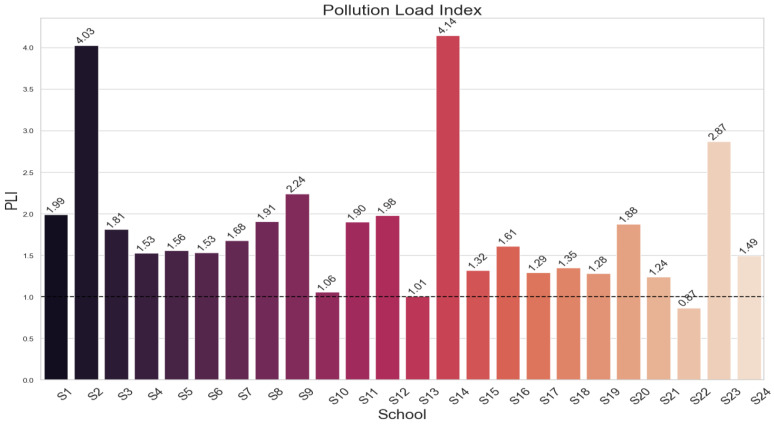
Pollution load index levels for each school.

The results of the contamination factor (CF) are shown in Figure 3. The CF values for Zn, Cu, and As showed a range of contamination levels, from very high to moderate (1.82–27.984). Zn was notably the most contaminated. Zr and Rb, on the other hand, showed little contamination. Significant contamination was found in a number of schools, especially in S2, S14, and S23, according to the modified contamination factor (mCF) study, which is shown in Figure 4. Figure 5’s Pollution Load Index (PLI) additionally identified schools with high pollution levels, specifically S2, S14, and S23, showing deteriorating environmental conditions in these locations.

### 3.3. Enrichment Factor

**Figure 6:**
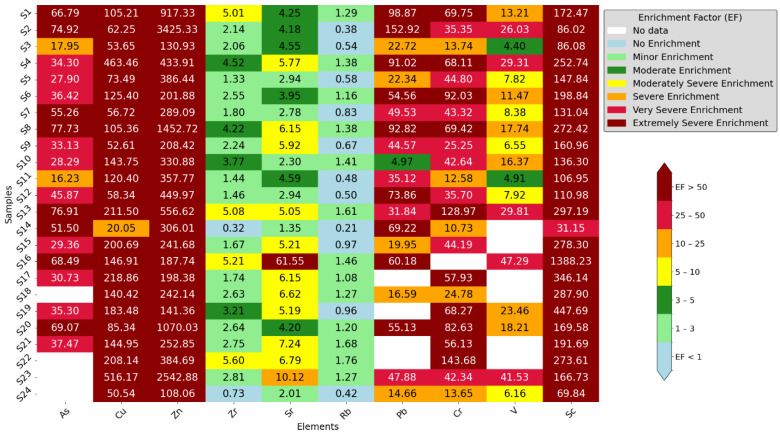
Enrichment values of samples from schools.

To evaluate the level of metal contamination across various schools, we calculated the enrichment factor (EF). Figure 6 depicts the enrichment factor of samples for each element across the areas. We found that most areas ranged from not being polluted to being extremely polluted. Notably, elements such as Cu, Zn, and Sc exhibited extremely severe enrichment, followed by As and Pb, which had above severe enrichments. Conversely, Zr and Rb had no enrichment to moderate enrichment. Of all the elements, Zn and Cr had the highest enrichment, as observed across all samples. Interestingly, similarities were observed across all school samples. 

### 3.4. Geo Accumulation Index 

**Figure 7:**
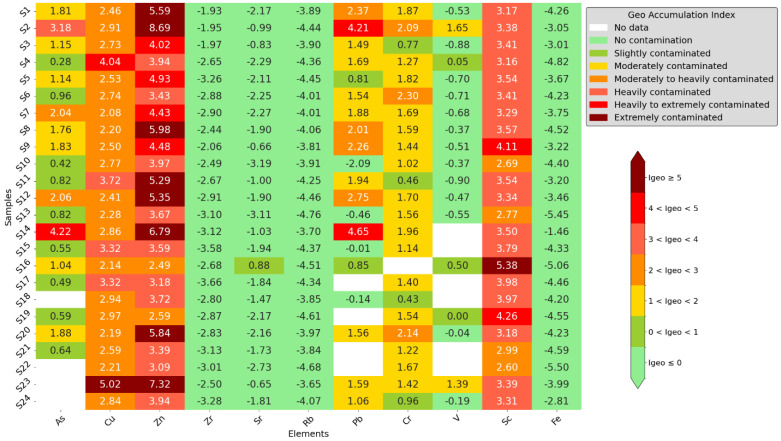
Geo-Accumulation Index results.

Figure 7 shows the Geo-Accumulation Index (I_geo_) heatmap, which shows different amounts of contamination in different samples. Zn was found to be highly contaminated (I_geo_ > 5) in multiple samples (1, 2, 8, 11, 12, 14, 20, and 23). I_geo_ readings for Pb, Cr, and As indicated important pollution, although not to as excessive an amount as Zn. Samples S23 and S16, however, revealed extreme Cu and Sc contamination. This analysis emphasizes how important it is to assess heavy metal pollution in environments such as schools using the Geo-Accumulation Index.

### 3.5. Pearson Correlation

**Figure 8:**
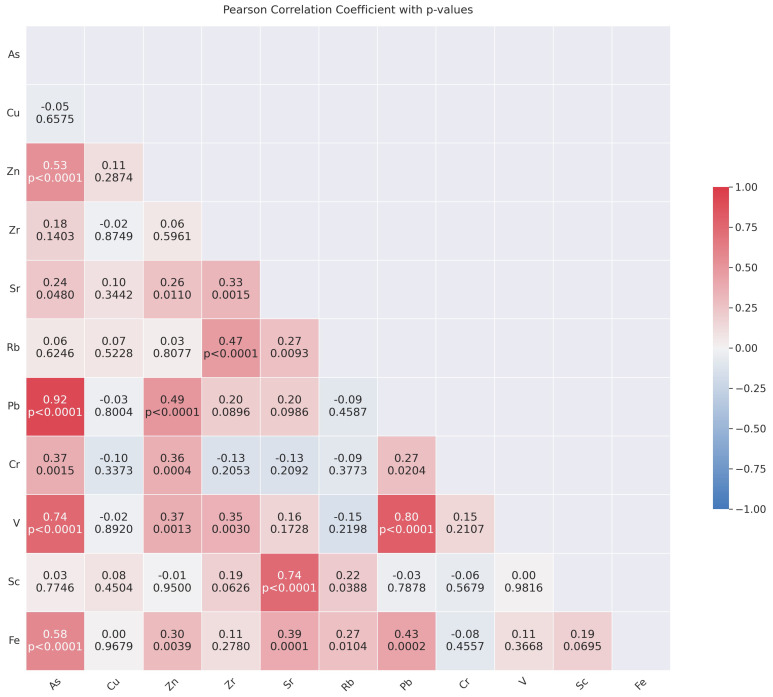
Pearson correlation coefficient matrix.

Significant positive correlations between different metal pairs are shown in the correlation matrix (Figure 8), which may indicate links between the metals’ presence in the samples. As and Pb, for instance, had a significant positive correlation of 0.92, suggesting that their sources or deposition processes may be similar. Other elements that had significant positive connections were Pb and V, and Sr and Sc, so on. Conversely, several element pairs showed weak to moderate correlations, indicating linkages that were not as strong. Comprehension of the complex interactions and common sources of these metals in educational environments requires a comprehension of these findings.

### 3.6. Principal Component Analysis 

**Figure 9:**
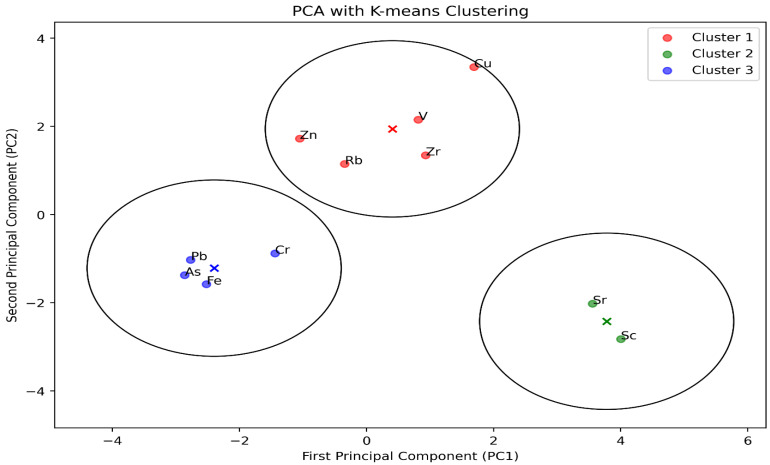
Principal components analysis.

One way to extract information on heavy metals is to use multivariate statistical methods, such as the principal component analysis (PCA) that Figure 9 illustrates. The correlation results showed a high complexity among the elements, which required further analysis to classify them and determine their origins [41]. PCA was an effective method to identify the pollution sources in this study. These techniques have been shown to be effective for this goal. PCA has been frequently used to detect pollution sources because it efficiently reduces the number of variables and so facilitates examination of the correlations between the observed variables. Significant correlations between heavy metal pairings, in general, indicate a common or combined origin, whereas weak correlations indicate separate origins [41].

A three-dimensional PCA figure shows more of the data variation than a two-dimensional one. For example, the three-dimensional PCA plot in this case explains about 70.2% of the total variance, while the two-dimensional plot explains only 51.6%. However, a three-dimensional PCA figure can also be harder to understand, so we decided to use and keep a two-dimensional PCA instead. Aside from dimensionality, many crucial factors impact the observed variance, such as environmental fluctuations at sampling sites, the age of buildings and building materials, temporal changes in pollutant levels in schools over time, and variations in sample characteristics. Data preprocessing, including normalization, also impacts the outcomes. These factors are crucial for a thorough comprehension of the variance explained by our PCA plots.

The elements showed distinct clustering patterns on the PCA plot, reflecting their correlations across the schools. K-means clustering confirmed three categories of elements after using elbow and silhouette methods to find optimal clusters. Cluster 1 consisted of Cu, Zn, Zr, Rb, and V, Cluster 2 of Sr and Sc, and Cluster 3 of As, Pb, Cr, and Fe. PCA and the cluster analysis revealed common origins or similar transport pathways for the elements that belong to the same cluster. This implies that the elements of Cluster 1 may have similar sources or undergo similar environmental processes as Clusters 2 and 3.

Given that all samples were collected from schools, and there were no municipal incineration or industrial sites involved in the city where the samples were taken, the similarities become even more notable. To fully understand why particular elements stand out, more research would be required. These differences can be the result of different sources for these elements or different distributional processes.

### 3.7. Hierarchical Clustering Analysis

**Figure 10:**
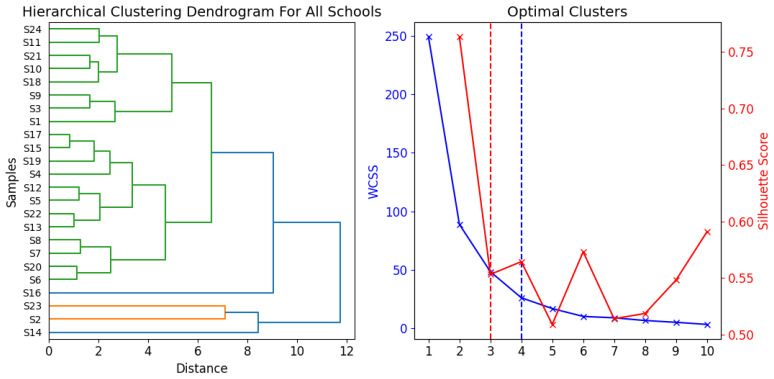
Optimal clusters and hierarchical clustering dendrograms for all sampled schools.

Figure 10 presents dendrograms and the optimal clusters for these dendrograms, derived through the elbow and silhouette methods. In our study, we employed the Euclidean distance to assess similarity. The hierarchical clustering analysis results, which incorporated the Ward linkage algorithm, are exhibited as dendrograms. 

**Figure 11:**
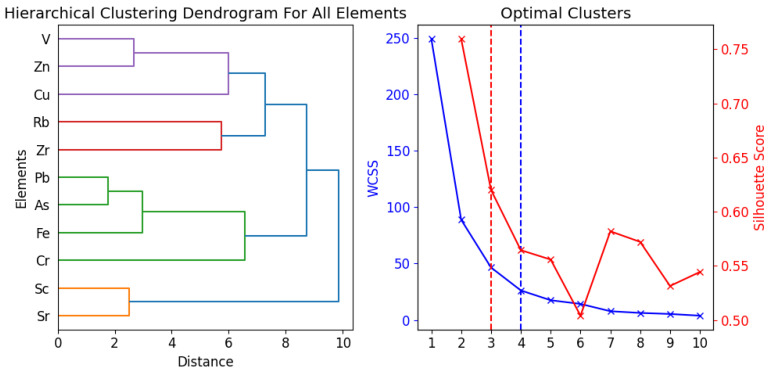
Optimal clusters and hierarchical clustering dendrograms for all sampled elements.

Figure 11 illustrates the similarities and dissimilarities for all school samples. Based on the optimal clusters, there are three main groups within the school samples. S2, S14 and S23 are placed in the first cluster, indicating that their compositions are quite similar; this also proves the corrected pollution assessments, where you could see higher pollutions. S16, which stands out from the other samples, was given its own cluster by the clustering algorithm and, along with the remaining samples, was split into three main groups, each containing various subgroups. Also, in Figure 11, we treated all school samples as a single entity and constructed a dendrogram for each element. We made the decision to separate our data into three unique clusters. The clustering algorithm’s findings showed the following groupings: Sr, Sc was in Cluster 1. As, Pb, Cr, and Fe were part of Cluster 2, while Cu, Zn, Zr, Rb, and V created Cluster 3. The samples within each cluster exhibited similar patterns of distribution for some elements. For example, Sr and Sc in Cluster 1 had consistent patterns across samples, suggesting that they might be influenced by the same factors or operations. The elements in Clusters 2 and 3 also showed comparable distributions to each other, but different from those of the elements in the other clusters. This could indicate multiple sources or processes affecting how these elements are distributed. 

The comparison between PCA and HCA clustering revealed identical findings with the same cluster groups. This enhances the robustness of our results and the interpretability of these clusters, as it suggests that the elements of each cluster may originate from the same location or be affected by the same anthropogenic or environmental factors.

Road dust contamination is largely attributed to tire wear, brake lining and road surface abrasion, leading to the presence of various heavy metals [46,47]. Metals stemming from vehicles, such as Cu, Zn, Cd, and Pb, primarily originate from wear and tear rather than combustion processes [48]. Specifically, asphalt and sandpaper-like effects contribute to As levels; As can also increase in the environment through the use of arsenic-based pesticides. The heavy metal concentrations in road dust are significantly affected by vehicle operation and road type. Brake dust, containing elements like Fe, Zn, Pb, Cu, and Cr, further adds to the contamination, with Cu and Cr being key tracers of non-exhaust brake and tire wear emissions [47,48]. Road travel has historically been a major source of Pb emissions, notably in Europe, where lead gasoline was widely used. The 2010 lead gasoline ban is expected to result in a considerable reduction in airborne Pb emissions [41,46].

The absence of proper ventilation, combined with the increased use of products like batteries, cell phones, and lights, worsens indoor air quality by limiting pollutant dilution [49]. Many batteries exceed EU limits for mercury and cadmium content, and, frequently, those containing excessive levels of mercury and/or lead are not properly labeled [50]. Furthermore, compact fluorescent lamps (CFLs) and LED bulbs are found to have high concentrations of Cu, Pb, and other heavy metals, posing risks to indoor air quality [51].

Elements like As, Cu, Zn, Pb, Cr, and Sc are likely dominated by anthropogenic input, which aligns with their relatively high enrichment factor (EF) values, indicating pollution in school samples. This pollution is likely due to the proximity of schools to roads, highways, and railways. To understand this more fully, we must examine the locations, pathways, and potential exposures within these schools.

### 3.8. Source Apportionment of Metals Using PMF

**Figure 12:**
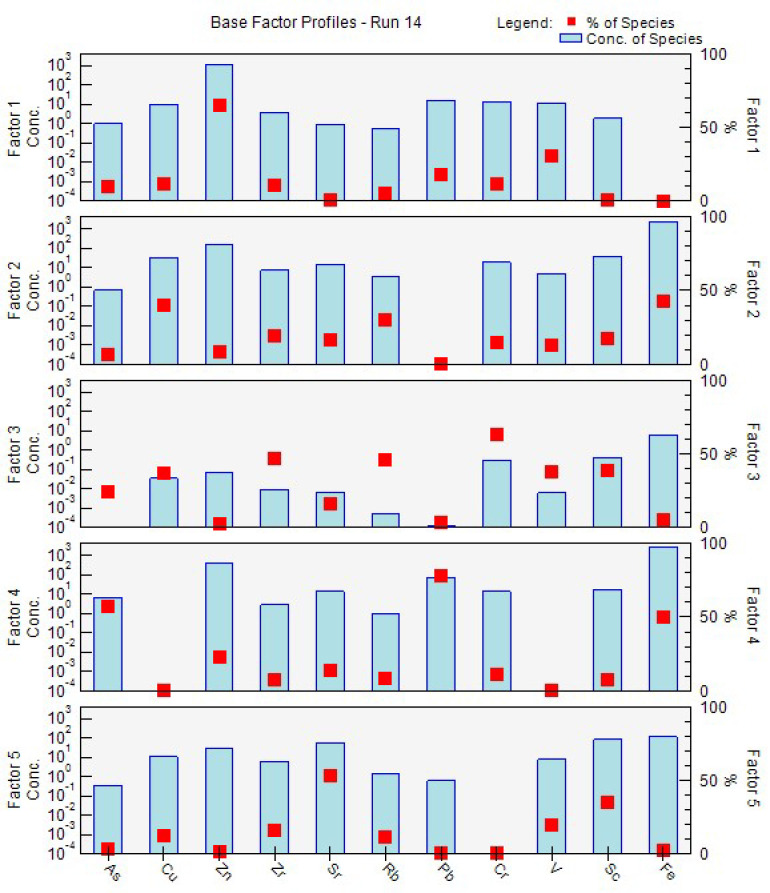
PMF factors from EPA application.

Figure 12 shows factors, and Factor 1 mostly represents Zn with contribution of V, Pb, Cr, Cu and As. Zn can be released into the environment through various sources such as electronics, construction, vehicular exhaust and tire debris, road dust, fossil fuel burning, and industrial gases [43,44]. Zinc-based products are commonly used as a coating or plating substance to protect other metals against corrosion. This is widespread in the construction industry, as steel structures are frequently zinc-coated to avoid rusting [52]. Hence, materials used in schools can contribute to a high level of Zn. 

Factor 2 described the significant loading of Fe and Cu. Fe can be released into the environment from various sources, including cars, industrial activities, fossil fuel combustion, and waste disposal. In cars, Fe is commonly used in brake pads and discs, and as these parts wear down, iron particles can be released. Iron is also present in the exhaust system of cars, including in the exhaust pipes and catalytic converters. Industrial activities such as iron and steel production, metalworking, and welding can also release iron into the environment [44,53]. Road dust particles containing Fe from nearby roads and highways, emissions from nearby train stations and iron-containing waste products can release Fe into the environment through waste disposal. Fossil fuel combustion is another source of Fe emissions [44]. Cu is commonly used in Cu–brass automotive radiators because of its high thermal conductivity and resistance to corrosion. It is also present in car lubricants. As the mechanical parts of vehicles degrade over time, copper is emitted into the surrounding environment [54], or industrial gasses [44].

Factor 3 was mostly influenced by Cr, Zr, Rb, and the contribution of Cu, As, Sc and V. Factor 3 can be released into the environment through industrial processes such as mining, smelting coal and oil combustion, the migration of rainwater through soil cracks, road dust emissions, the wear and tear of asbestos linings and cement dust, as well as Cr-coated metals and waste disposal sites like landfills and hazardous waste sites. In heavy traffic road dust, Cr was identified because of brake linings [43,55]. Anthropogenic sources of Zr include the manufacturing and utilization of zirconium-based products like paints, ceramics, alloys, catalytic converters in new cars and refractory bricks, as well as the disposal of these products [56]. Rb is widely present in the Earth’s crust and its anthropogenic sources include the manufacturing and utilization of rubidium-based compounds in various industries, as well as the disposal of these compounds and coal burning [57].

Factor 4 provided insight for the high loadings of Pb, As and Fe (metal processing, coal-fired power generation, wood preservatives, waste disposal reasons). Dust particles containing Pb and As can originate from nearby road dust, as well as ongoing construction activities. Anthropogenic As deposition is mainly caused by atmospheric pollution and the use of phosphate fertilizers; this could be the cause of weather conditions. Smoke emitted from industrial units or power plants can carry metal particles that settle on the ground and contribute to road dust. These metal particles eventually become suspended in the air and settle on the ground, joining the existing dust particles [43,58]. Lead-based paint found on aging school structures and the degradation of building materials contribute to the presence of Pb in the environment; Lithuania was one of the largest exporters of paints by volume in 2008 and 2009 in Europe [59]. Also, Pb were identified among the heavy traffic road dust [55]. 

Factor 5 is characterized by a strong association with Sr, Sc and with the contribution of Rb and V. The production and disposal of electronic devices, particularly fluorescent lamps, can contribute to elevated levels of Sr in dust. This is particularly relevant in schools where fluorescent lamps are commonly used, and broken lamps can release even higher concentrations of Sr [60]. V can be derived from domestic heating and automotive traffic [61]. The major application of Sc is as an alloy with metals like Al, Mg, Zr; in our case, this could be the reason [62].

It is important to mention that urban road dust is associated with road traffic, which combines contributions of vehicular non-exhaust emissions (tire wear and brake abrasion) and road pavement/furniture [44]. Furthermore, all the schools in the Vilnius area are located in close proximity to various sources of potential pollution. While there are no prominent industrial zones, the presence of power plants, roads, highways, and train stations suggests these as potential pollution sources. Additionally, certain elements may be transported through wind dispersion, while local sediment can also contribute to pollution levels.

The results of the hierarchical cluster analysis (HCA) may align with the results of the principal component analysis (PCA) and positive matrix factorization (PMF). Techniques such as correlation matrix analysis, PCA, PMF and HCA are commonly used to identify the sources of heavy metal pollution in indoor dust. Also, the combination of Pearson correlation, PCA, HCA, and PMF studies reveals regular patterns in the interactions between distinct variables, helping to identify common causes or similar environmental behaviors. For example, the high positive correlations between elements such as As and Pb, Zn and V, and Sr and Sc and so on, together with their clustering in the same PMF factors, imply common sources or similar transit and deposition processes. 

### 3.9. Particulate Matter Ratio

**Figure 13:**
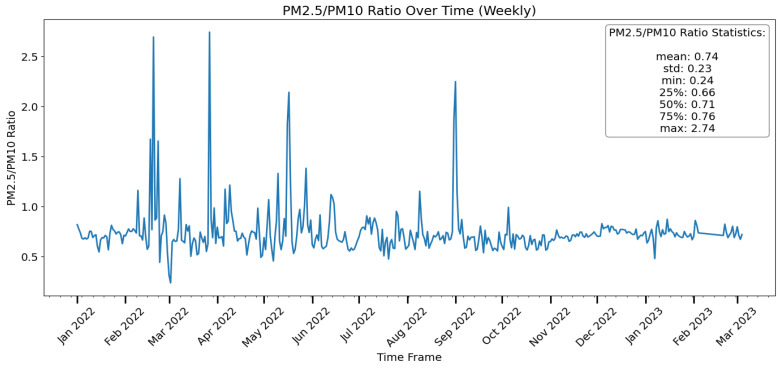
PM 2.5 / PM10 ratio over a year in Vilnius city (Vilnius Municipality, 2023).

**Figure 14:**
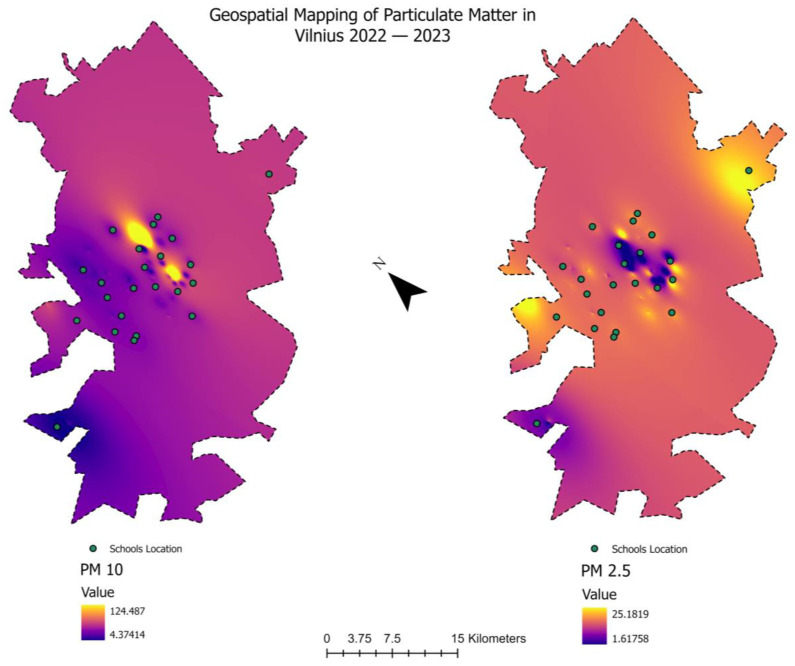
PM 2.5 and PM10 between 2022–2023 in Vilnius city [66].

Figure 13 and Figure 14 show a clear trend of an increasing PM2.5/PM10 ratio over time. PM2.5/PM10 ratios can provide a series of important information such as the cause of pollution, the air pollution process, and the impact on life and health. Generally, a lower PM2.5/PM10 ratio indicates coarse particles are dominant, which is more attributed to natural sources. In contrast, higher PM2.5/PM10 ratios indicate that the air pollution is more from anthropogenic sources [63]. Larger ratios indicate more combustion-related pollution (such as car exhaust or burning), and lower ratios indicate more mechanical or natural causes (such as dust or pollen).

In Europe, with a higher level of urbanization, the PM2.5/PM10 ratios are between 0.39 and 0.74, with the lowest values occurring in Southern Europe and the highest values occurring in Eastern Europe [64]. A regional study of mixed agricultural and industrial development in the state of São Paulo, Brazil, showed that the PM2.5/PM10 ratios were in the range of 0.33–0.47. In Saudi Arabia in the Middle East, which is obviously affected by the desert and arid climate, the PM2.5/PM10 ratios were 0.25–0.52, and the average value was only 0.3 [63].

The graph also reveals some variation in the ratio, with certain periods of time having a larger ratio than others. While the effects of seasonality, vehicular emissions, and residential heating significantly influence the ratios of PM2.5 to PM10, we cannot disregard the role of weather conditions. Even if the pollution source remains the same, variations in weather can lead to substantial differences in pollution levels [65]. The PM2.5/PM10 ratio is often greater than 0.5, which is considered high. This means that the air quality in the area is likely to be poor, which could have negative health effects, particularly for persons with respiratory disorders. The graph reveals some ratio peaks in the time frame. This could point to events or periods with exceptionally high levels of combustion-related pollution. These extreme values highlight the possibility of instances in which the ratio deviates greatly from the average, demonstrating the impact of unique emission sources or environmental variables on particle composition.

**Figure 15:**
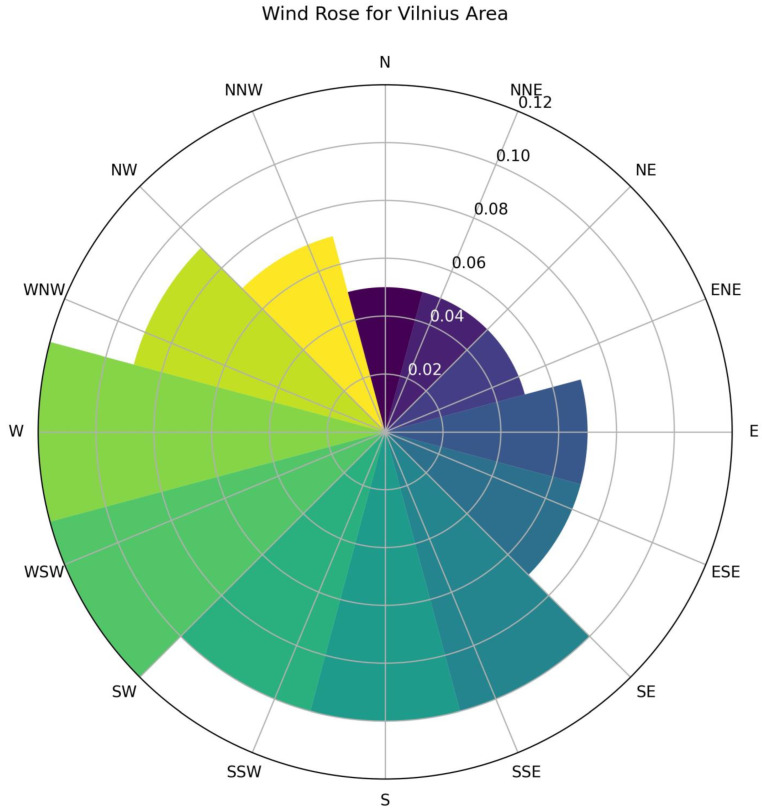
Wind rose for Vilnius area [67].

Figure 15 illustrates the wind rose for Vilnius, indicating potential routes through which winds could transport pollution. Therefore, the PM2.5/PM10 ratios are affected by multiple factors such as the underlying surface, human activities, possibly regional or long-range transport and meteorological conditions, resulting in large spatial–temporal variations [63].

### 3.10. Hazard Index for Health Risk

**Figure 16:**
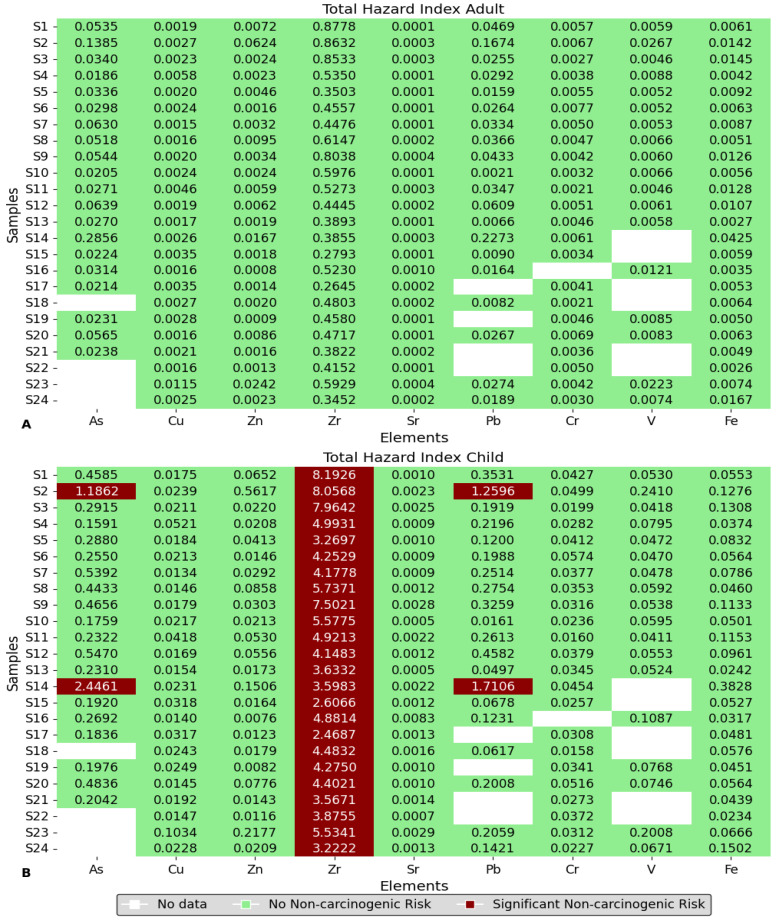
Hazard index values for children and adults in sampled schools for each element.

Figure 16 presents the total hazard index (HI) for both adults and children. A value above 1 signifies a non-carcinogenic health risk. Graph A displays the results for adults, with all values falling below 1. Additionally, As and Pb present a health risk for children in schools S2 and S14. All other elements have values below 1, indicating no non-carcinogenic risk. 

However, Graph B, which represents the child HI, shows that for Zr, all values exceed 1. The Zr content in the adult human body is about 420 mg [68]. It is important to consider common Zr-containing products, especially in laboratory equipment and ceramic components of electronic devices [69], paper coatings [70], knives, scissors, and golf irons due to their strong and biocompatible nature [71]. Authorities should regulate the use and disposal of zirconium-containing products to minimize exposure to zirconium pollution. This may involve implementing appropriate waste management procedures for zirconium-containing items in schools and conducting frequent environmental health assessments to monitor zirconium levels in the air and dust.

Metal concentrations in various schools were found to vary significantly, highlighting the need to consider location, construction materials, and environmental conditions when measuring metal deposition. The presence of large amounts of metals on the school premises raises issues about children’s expected safety and health standards.

According to the study findings, many of the schools were only a few meters away from frequently used roadways, and some schools were close to train stations, implying that motor vehicles emissions, lubricating oil and grease, and the abrasive wear of rail tracks were most likely the primary source of these metals. The finding implies that pollution from motor vehicles may have had a major impact on the presence of these metals in schools. Additionally, metal concentrations may have been influenced by emissions from surrounding restaurants and residential areas, as well as the pollution from power plants through burning fuels. 

## 4. Limitations

This study, examining the presence of heavy metal contamination in dust within schools in Vilnius, provides valuable insights. However, it is subject to various limitations. The variety of schools available in Vilnius may not fully represent the complete spectrum of educational environments, which could impact the generalizability of the findings. The methodology for collecting and analyzing dust samples, despite its rigor, faces limitations in sample contamination risks and the sensitivity of analytical methods, which could impact the accuracy of metal concentration measurements. The main aim of this study was to evaluate the long-term accumulation of dust and heavy metal pollution in the school environment. However, it did not particularly investigate any changes that occur with the seasons. Gaining comprehension of these seasonal variations could offer supplementary understanding of the temporal patterns of dust and heavy metal accumulation. Hence, although our discoveries provide significant insights into the amounts of pollution over an extended period, doing further studies that incorporate a seasonal analysis could augment our comprehension of these environmental elements. The health risk assessment, relying on specific assumptions and models, may not comprehensively represent complex real-world exposure scenarios, thereby making the projected risks indicative rather than definitive. Furthermore, it is important to use caution when applying these findings to other situations, taking into account the distinct geographical, environmental, and socio-economic aspects of the schools that were studied. Recognizing these constraints is essential for presenting a balanced perspective on the possible presence of heavy metal pollution in educational environments and its impact on children’s health. Further investigation is required to overcome these limitations and gain a more thorough understanding of the situation.

Strategies for Reducing Exposures and Mitigating Heavy Metal Concentrations

- Enhanced cleaning protocols involve implementing strict cleaning routines to constantly eliminate dust and particle debris that may accumulate heavy metals. Areas with strong student activity require special care for policy reforms that mandate the implementation of best practices in cleaning and maintenance within schools to minimize exposure to heavy metals. This could include guidelines for cleaning methods that reduce the resuspension of dust particles and the use of cleaning products that do not contribute to indoor pollution.
- To mitigate the inhalation risks associated with contaminated dust, regulations or guidelines could be developed to mandate the installation and maintenance of high-efficiency particulate air (HEPA) filters in school ventilation systems. Installing high-efficiency filters in school ventilation systems can effectively collect airborne particles and limit the risk of inhaling contaminated dust [72,73]. These policies could outline specific performance standards for filters based on the local environmental context and the unique needs of educational facilities.
- Green infrastructure involves using green spaces like gardens and green roofs around school buildings to serve as natural filters for air pollutants, decreasing the infiltration of outdoor pollution into inside areas [74,75].
- Developing educational programs to teach students and staff about environmental health risks and preventive activities to promote a culture of safety and awareness.
- Policies promoting collaboration between schools, municipal authorities, environmental agencies, and community organizations can lead to comprehensive approaches to tackle environmental pollution sources. Such policies could establish frameworks for shared responsibility and action, including pollution monitoring, community awareness programs, and the implementation of local pollution control measures.
- Mandatory health and safety audits, including environmental health assessments in schools, can identify and manage heavy metal pollution sources. Policies could require regular audits by certified environmental health professionals to assess the levels of heavy metals in school environments and recommend mitigation measures. These audits could be supported by a central database managed by educational or environmental health authorities to track pollution levels and mitigation efforts over time. Establishing clear guidelines for these assessments, including frequency, methods, and follow-up actions, will be crucial for their success.
- Countries planning school renovations should adopt regulations for the effective removal and management of accumulated dust, leveraging insights from this study to minimize heavy metal exposure risks. Sharing best practices on heavy metal dust mitigation across borders can guide the implementation of safer renovation protocols, ensuring educational environments worldwide are protected from contamination.

## 5. Conclusions

This study offers a pivotal examination of metal contamination in the indoor dust found in schools in Vilnius, which is an area that has not been extensively investigated in Lithuanian research. The results of our study support the idea that there is a higher presence of heavy metals in the dust, which mostly originates from different sources of pollution, both external and internal. Significant variations were seen in elements such as arsenic (As), copper (Cu), zinc (Zn), and lead (Pb) among different schools, indicating the influence of local environmental conditions and human activities. The utilization of advanced statistical techniques, see Table S3 from Supplementary Materials, that underscore the complex interplay of pollution sources affecting school environments included hierarchical cluster analysis, principal component analysis, and positive matrix factorization, which played a crucial role in the identification of these sources of contamination and their potential health hazards, particularly for children. The study highlights the urgent requirement for efficient pollution control methods in school environments, as indicated by the elevated hazard index of hazardous elements such as Pb and As. Our research provides valuable information about the composition and sources of dust pollution in educational environments. However, it also emphasizes the necessity for wider geographical sampling and longer-term data collecting to address some limitations. Further studies should prioritize enhancing our comprehension of pollution origins and formulating precise measures to protect the well-being of students in Vilnius and comparable metropolitan regions. Our research suggests that implementing a comprehensive strategy involving improved cleaning procedures, high-efficiency ventilation filters, green infrastructure, educational initiatives, community involvement in pollution control, and regular environmental health evaluations can effectively decrease heavy metal exposure in schools, thus creating a safer educational setting.

## Figures and Tables

**Table 1:** Exposure parameters used for the health risk assessment through different exposure pathways for dust [36,37,38].

	Parameters and Units	Child	Adult
C	Concentration of the element (mg/kg)		
IngR	the ingestion rate (mg/day)	200	100
SA	the surface area of the skin exposed to heavy metals (cm^2^)	2800	5700
AF	the skin adherence factor (mg/cm^2^);	0.2	0.7
ABS	dermal absorption factor (unitless)	0.001	0.001
InhR	the inhalation rate (m^3^/day);	7.6	20
PEF	the particle emission factor (m^3^/kg)	1.4 × 10^9^	1.4 × 10^9^
EF	the exposure frequency (days/year);	285	285
ED	the exposure duration (year);	6	30
BW	the body weight (kg)	15	70
AT	the average time (days);		
	For carcinogens	25,550	25,550
	For non-carcinogens	2190	10,950
CF	the conversion factor	1 × 10^−6^	1 × 10^−6^
VF	volatilization factor m^3^/kg	32,675.6	32,675.6

**Table 2:** Used RfD values.

Element	RfD Ingestion	RfD Dermal	RfD Inhalation
As	0.0003	0.000123	0.000301
Cu	0.04	0.0402	0.012
Zn	0.3	0.3	0.35
Zr	0.00008	-	-
Sr	0.6	0.12	0.6
Pb	0.0035	0.00053	0.0035
Cr	1.5	0.006	0.00003
V	0.007	0.007	0.00007
Fe	0.7	0.7	0.8

## Data Availability

The data that support the study’s findings are not publicly available owing to sensitivity concerns but are available from the corresponding author upon reasonable request.

## Supplementary Materials

The following supporting information can be downloaded at: https://www.mdpi.com/article/10.3390/toxics12030224/s1, References [76,77] are cited in the Supplementary Material. Table S1: Mean concentration of sampled schools in mg/kg ; Table S2: Dust concentrations found in similar research.; Table S3: Potential pollution sources for HCA, PCA and PMF factors.
