# Supplementary material for: Assessment of Heavy Metal Contamination in Dust in Vilnius Schools: Source Identification, Pollution Levels, and Potential Health Risks for Children
Source: Toxics. 2024 Mar 19;12(3):224. doi: 10.3390/toxics12030224 (PMC10974985; doi:10.3390/toxics12030224)
Supplement: Supplementary file 1 [file toxics-12-00224-s001.zip › toxics-2881288-supplementary.pdf]

Assessment of Heavy Metal Contamination in Dust in Vilnius Schools: Source Identification, Pollution Levels, and Potential Health Risks for Children

Murat Huseyin Unsal [1,*], Gytautas Ignatavičius [1], Arunas Valiulis [2,3], Nina Prokopciuk [2], Roberta Valskienė [4] and Vaidotas Valskys [1,5]

1 Institute of Biosciences, Life Sciences Center, Vilnius University, Saul˙ etekio Ave. 7, 10257 Vilnius, Lithuania; gytautas.ignatavicius@gf.vu.lt (G.I.); vaidotas.valskys@gmc.vu.lt (V.V.)

2 Clinic of Children's Diseases, Institute of Clinical Medicine, Medical Faculty, Vilnius University, Antakalnio St. 57, 10207 Vilnius, Lithuania

3 Department of Public Health, Institute of Health Sciences, Medical Faculty, Vilnius University, M. K. Cˇ iurlionio St. 21, 03101 Vilnius, Lithuania

4 Nature Research Centre, Laboratory of Ecotoxicology, Akademijos St. 2, 08412 Vilnius, Lithuania; roberta.valskiene@gamtc.lt

5 Nature Research Centre, Laboratory of Climate and Water Research, Akademijos St. 2, 08412 Vilnius, Lithuania

* Correspondence: murat.unsal@gmc.stud.vu.lt

Table S1: Mean concentration of sampled schools in mg/kg

mg/kg	As	Cu	Zn	Zr	Sr	Rb	Pb	Cr	V	Sc	Fe
**S1**	13.11	66.93	1873.24	62.95	56.70	14.21	116.48	164.35	33.19	176.10	3707.10
**S2**	33.93	91.33	16131.34	61.91	128.65	9.66	415.47	192.09	150.86	202.56	8549.42
**S3**	8.34	80.72	632.33	61.20	143.53	14.07	63.30	76.56	26.17	207.88	8767.57
**S4**	4.55	199.18	598.57	38.37	52.04	10.25	72.44	108.41	49.77	174.32	2504.30
**S5**	8.24	70.31	1186.70	25.13	58.97	9.61	39.57	158.74	29.55	226.99	5574.71
**S6**	7.29	81.37	420.50	32.68	53.76	13.06	65.56	221.18	29.41	207.08	3781.25
**S7**	15.42	51.28	839.02	32.10	52.80	13.00	82.94	145.07	29.93	190.16	5268.82
**S8**	12.68	55.68	2464.17	44.09	68.22	12.62	90.84	135.88	37.05	231.05	3079.35
**S9**	13.32	68.52	871.25	57.65	161.86	15.01	107.50	121.79	33.70	336.43	7588.79
**S10**	5.03	82.83	611.95	42.86	27.85	14.00	5.30	90.99	37.26	126.04	3357.46
**S11**	6.64	159.60	1522.27	37.82	127.58	11.04	86.20	61.78	25.70	227.53	7724.22
**S12**	15.64	64.46	1595.86	31.88	68.24	9.53	151.13	146.11	34.59	196.80	6438.40
**S13**	6.61	58.87	497.30	27.92	29.51	7.76	16.41	132.96	32.78	132.76	1621.90
**S14**	69.96	88.26	4323.60	27.65	124.73	16.12	564.25	175.00	<BDL	220.03	25649.29
**S15**	5.49	121.60	470.05	20.03	66.24	10.16	22.38	99.16	<BDL	270.63	3530.75
**S16**	7.70	53.51	219.50	37.51	470.53	9.22	40.59	<BDL	68.05	811.54	2122.49
**S17**	5.25	121.13	352.42	18.97	71.38	10.34	<BDL	118.75	<BDL	307.46	3225.05
**S18**	<BDL	93.04	514.97	34.45	92.09	14.58	20.36	60.82	<BDL	306.15	3860.89
**S19**	5.65	95.16	235.32	32.85	56.53	8.58	<BDL	131.14	48.06	372.64	3022.12
**S20**	13.83	55.36	2228.31	33.83	57.17	13.42	66.23	198.55	46.68	176.58	3780.50
**S21**	5.84	73.20	409.87	27.41	76.72	14.65	<BDL	104.99	<BDL	155.36	2942.69
**S22**	<BDL	56.05	332.52	29.78	38.38	8.18	<BDL	143.30	<BDL	118.25	1569.18
**S23**	<BDL	395.37	6252.00	42.53	162.70	16.77	67.91	120.13	125.67	204.97	4463.36
**S24**	<BDL	87.28	599.02	24.76	72.72	12.53	46.88	87.28	42.01	193.58	10063.49

BDL = Below Detection Limit

Table S2: Dust concentrations found in similar research.

Locations	Cr	Cu	Zn	Pb	Fe	As	References
**Malaysia, Indoor dust**	16.88	30.19	148.71	31.24	4225.33	-	[2]
**Iraq, Indoor dust**	65.68	54.28	43.90	51.46	-	-	[3]
**Nigeria, Indoor dust**	41.80	12.70	121.00	27.60	13.70	2.04	[13]
**Hong Kong, Indoor and Outdoor dust**	-	247.38	2293.56	199.96	-	-	[14]
**Ghana, Indoor dust**	381.30	-	-	4.82	-	-	[15]
**Istanbul, Outdoor dust**	254.00	513.00	1970.00	192.00	-	-	[18]
**Istanbul, Outdoor dust**	89.00	200.00	984.00	30.00	-	-	
**Warsaw, Outdoor dust**	90.00	109.00	1070.00	124.00	-	-	
**New Zealand, Indoor dust**	-	-	21700.00	724.00	-	-	
**South Africa, Outdoor dust:**							[76]
**School A**	87.90	38.00	148.70	12.45	-	2.90	
**School B**	37.13	60.97	107.35	15.86	-	0.78	
**School C**	57.45	7.78	45.10	8.08	-	1.66	
**School D**	82.40	28.66	315.10	52.68	-	1.60	
**School E**	45.60	44.91	9.53	16.49	-	2.07	
**School F**	34.90	116.80	37.50	24.43	-	0.99	
**School G**	24.35	66.85	37.90	62.85	-	1.82	
**School H**	27.15	416.65	41.10	184.20	-	0.96	
**Greece, Outdoor dust**	87.00	-	1505.00	133.00	-	-	[77]
**Sydney, Indoor dust**	90.00	-	1876.00	299.00	-	17.60	
**Ottawa, Indoor dust**	86.70	206.00	717.00	406.00	-	7.30	[19]
**Canada, Indoor dust**	117.00	279.00	833.00	210.00	-	13.10	
**USA, Indoor dust**	-	-	876.00	109.00	-	6.30	
**Sydney, Indoor dust**	65.00	-	372.00	76.00	2790.00	-	
**China, Indoor and Outdoor dust**	149.20	70.80	461.50	180.90	-	13.20	[21]
**Hermosillo, Outdoor dust**	-	26.34	387.98	36.15	-	-	[40]
**Iran, indoor dust:**							[20]
**Cold Season**	67.00	158.00	513.00	56.00	-	-	
**Warm Season**	97.00	127.60	666.70	292.00	-	-	
**Tokyo and Hiroshima**	67.80	304.00	920.00	57.90	-	-	

Table S3 Potential pollution sources for HCA, PCA and PMF factors

Analysis Method	Identified Elements	Potential Pollution Sources
**HCA**	Cluster 1: Sr and Sc<br>Cluster 2: As, Pb, Cr, and Fe	Urban road dust (vehicular non-exhaust emissions, road

	Cluster 3: Cu, Zn, Zr, Rb, and V	pavement/furniture), power plants, roads, highways, train stations, wind dispersion, local sediment
**PCA**	Cluster 1: Sr and Sc<br>Cluster 2: As, Pb, Cr, and Fe<br>Cluster 3: Cu, Zn, Zr, Rb, and V	Similar patterns across all elements suggest common sources as HCA
**PMF Factor 1**	Zn, V, Pb, Cr, Cu, As	Electronics, construction, vehicular exhaust and tires debris, road dust, fossil fuel burning, industrial gases, zinc-coated materials in schools
**PMF Factor 2**	Fe, Cu	Cars (brake pads, discs, exhaust system), industrial activities, fossil fuel combustion, waste disposal, road dust, train station emissions, copper-brass automotive radiators, car lubricants
**PMF Factor 3**	Cr, Zr, Rb, Cu, As, Sc, V	Coal and oil combustion, rainwater migration through soil, road dust emissions, wear and tear of asbestos linings and cement dust, Cr-coated metals, waste disposal sites, manufacturing and utilization of zirconium-based products, rubidium-based compounds
**PMF Factor 4**	Pb, As, Fe	Coal-fired power generation, wood preservatives, waste disposal, road dust, construction activities, atmospheric pollution, phosphate fertilizers, lead-based paint on aging school structures, degradation of building materials
**PMF Factor 5**	Sr, Sc, Rb, V	Production and disposal of electronic devices (particularly fluorescent lamps), domestic heating, automotive traffic, alloy production with metals
